# Evolution of Resting Energy Expenditure, Respiratory Quotient, and Adiposity in Infants Recovering from Corrective Surgery of Major Congenital Gastrointestinal Tract Anomalies: A Cohort Study

**DOI:** 10.3390/nu12103093

**Published:** 2020-10-11

**Authors:** Luís Pereira-da-Silva, Susana Barradas, Ana Catarina Moreira, Marta Alves, Ana Luisa Papoila, Daniel Virella, Gonçalo Cordeiro-Ferreira

**Affiliations:** 1 NOVA Medical School, Faculdade de Ciências Médicas, Universidade Nova de Lisboa, Campo dos Mártires da Pátria, Number 130, 1169-056 Lisbon, Portugal; ana.papoila@nms.unl.pt (A.L.P); goncalo.cf@chlc.min-saude.pt (G.C.-F.); 2 Neonatal Intensive Care Unit, Hospital Dona Estefânia, Centro Hospitalar Universitário de Lisboa Central, Rua Jacinta Marto, 1169-045 Lisbon, Portugal; danielvirella@chlc.min-saude.pt; 3 Nutrition Lab, Department of Pediatrics, Hospital Dona Estefânia, Centro Hospitalar de Lisboa Central, Rua Jacinta Marto, 1169-045 Lisbon, Portugal; 4 Research Unit, Centro Hospitalar Universitário de Lisboa Central, Rua Jacinta Marto, 1169-045 Lisbon, Portugal; marta.alves@chlc.min-saude.pt; 5 Dietetics and Nutrition, Lisbon School of Health Technology, Av. Dom João II MB, 1990-094 Lisbon, Portugal; ana.moreira@estesl.ipl.pt; 6 MSc Program, Faculdade de Medicina de Lisboa and Lisbon School of Health Technology, Av. Dom João II MB, 1990-094 Lisbon, Portugal; susana.isabel.barradas@marinha.pt

**Keywords:** adiposity, congenital gastrointestinal anomalies, fat mass index, infants, respiratory quotient, resting energy expenditure

## Abstract

This cohort study describes the evolution of resting energy expenditure (REE), respiratory quotient (RQ), and adiposity in infants recovering from corrective surgery of major congenital gastrointestinal tract anomalies. Energy and macronutrient intakes were assessed. The REE and RQ were assessed by indirect calorimetry, and fat mass index (FMI) was assessed by air displacement plethysmography. Longitudinal variations over time are described. Explanatory models for REE, RQ, and adiposity were obtained by multiple linear regression analysis. Twenty-nine infants were included, 15 born preterm and 14 at term, with median gestational age of 35.3 and 38.1 weeks and birth weight of 2304 g and 2935 g, respectively. In preterm infants, median REE varied between 55.7 and 67.4 Kcal/kg/d and median RQ increased from 0.70 to 0.86–0.92. In term infants, median REE varied between 57.3 and 67.9 Kcal/kg/d and median RQ increased from 0.63 to 0.84–0.88. Weight gain velocity was slower in term than preterm infants. FMI, assessed in a subset of 15 infants, varied between a median of 1.7 and 1.8 kg/m^2^ at term age. This low adiposity may be related to poor energy balance, low fat intakes, and low RQ¸ that were frequently recorded in several follow-up periods.

## 1. Introduction

### 1.1. Determinants of Resting Energy Expenditure in Infants

In neonates and young children, the organs grow in proportion to body weight [1], but information on mass-specific metabolic rate of their faster growing organs and tissues is limited and has not been sufficiently studied.

In infants, factors other than body mass, contribute significantly to resting energy expenditure (REE), including gestational age, postnatal age, body weight gain, and energy intake [2,3]. In infants born preterm and in sick infants, REE is also affected by the metabolic stress underlying the clinical condition, as well as body temperature, metabolic rate, and therapy with stimulant drugs such as caffeine and catecholamines [4,5,6]. As physical activity and thermic effects of food significantly affect REE, measurements of REE should ideally be undertaken with infants in deep sleep and during fasting [4,7].

Longitudinal reference values of REE are available for healthy preterm and full-term infants during the first postnatal weeks [3].

### 1.2. Energy and Macronutrient Needs in Surgical Neonates

The energy metabolism of neonates undergoing major surgery has been less studied than in healthy infants [8]. The metabolic response to trauma, such as major surgery, is characterized by three phases: A very early ebb phase with decreased metabolic rate, the early catabolic flow phase that compensates for the initial trauma, and the late anabolic phase after the metabolic response to trauma has ended that is characterized by gradual restoration of body protein and fat stores [9,10]. Most of the studies of metabolic response to surgical stress have focused on the early postsurgical phases [11,12,13,14] when infants are still dependent on parenteral nutrition and are still undergoing stress and dysregulation of metabolism [6,9,10]. Data on REE in the late anabolic phase, when infants are undergoing catch-up growth and are enterally fed, are scarce [15,16,17].

Nutritional management specifically for infants recovering from corrective surgery of major congenital anomalies is not well defined, and recommendations for energy and macronutrients intake are needed [18,19,20]. Surgical neonates may need extra energy supply to reduce fat oxidation and facilitate lipogenesis, considering that increased protein synthesis for tissues repair in postsurgical period is a high energy-requiring process [21]. Furthermore, the heterogeneity of underlying surgical conditions usually requires individualized nutritional regimens to face unique metabolic demands [22,23]. Studies of nutritional requirements of surgical neonates based on large homogenous sample sizes are difficult to obtain and grouped data analysis may not reflect individual variability [24].

The individualized nutritional regimen for surgical neonates may be better defined by the REE [12] and the respiratory quotient (RQ), provided by indirect calorimetry [7,25]. Portable indirect calorimetry is noninvasive, and has been validated for accuracy and repeatability in neonates, including sick or physiologically unstable preterm infants [26]. REE measurements can be useful guides to energy supply, and RQ measurements indicate the preferential substrate utilization that can help guide macronutrient supplies [7,25]. Interpretation of indirect calorimetry measurements can be improved when combined with longitudinal assessments of body composition changes, allowing insight into the effects of energy and different macronutrient intakes on growing fat mass FM and fat-free mass (FFM) [27,28] in preterm and term infants, including in surgical neonates [1,19]. Measurement of FM and FFM by air displacement plethysmography has been validated among stable infants [29,30], and it is noninvasive, rapid to perform, and not affected by body movements [31].

Pereira-da-Silva et al. (2015) [16] reported four infants with unexpected adiposity depletion in spite of satisfactory weight gain, with subsequent adiposity catch-up associated with high REE and RQ in the late anabolic phase after corrective surgery of major congenital gastrointestinal anomalies. These findings motivated the study of a larger sample of infants with similar surgical conditions, further exploring factors associated with the reported changes of adiposity.

### 1.3. Objective

This study was aimed at quantifying the evolution of REE, RQ, and adiposity of infants during the late anabolic phase after corrective surgery of major congenital gastrointestinal tract anomalies and to explore the determinants associated with these components of energy balance.

## 2. Materials and Methods

### 2.1. Design, Setting and Ethical Issues

This single-center, observational, longitudinal study was conducted for 18 months (July 2017 to December 2018) in the Neonatal Intensive Care Unit and the Nutrition Lab of Hospital Dona Estefânia, Centro Hospitalar Universitário de Lisboa Central. Institutional ethical committee approval (Nr 475/2017, dated 12th July 2018) and parental-informed consent were obtained.

### 2.2. Eligibility, Recruitment, Inclusion and Exclusion Criteria

Neonates subject to corrective surgery of major congenital gastrointestinal tract anomalies early after birth were eligible. Infants were recruited consecutively as soon as they were not dependent on supplemental oxygen. Only infants undergoing indirect calorimetry measurements during at least two consecutive weeks were included and followed up to discharge. Infants with associated conditions that increase metabolic rates, such as congenital heart disease complicated with a hyperdynamic circulatory state, or decrease metabolic rates, such as hypothyroidism, were excluded. The size of the convenience sample was determined by the observer’s availability (SB).

### 2.3. Nutritional Intake and Body Weight Records

Daily energy and macronutrient intakes by parenteral and enteral nutrition, type of feeding (fortified or non-fortified human milk, type of formula), and mode of administration (continuous or intermittent) were recorded from birth to discharge by the same investigator (SB). Following the unit protocol, if vomiting was considered excessive or daily gastric residuals were more than 50% of the intake in the previous 24 h, energy and macronutrients administered by enteral route were not took into account. Routine body weight, daily measured by nurses using scales incorporated in incubators or external automatic scales, was daily recorded.

### 2.4. Indirect Calorimetry Measurements

As previously described [16], REE and RQ were assessed using the portable open-circuit continuous indirect calorimetry Deltatrac II Metabolic Monitor (Datex-Ohmeda, Instrumentarium Corp, Helsinki, Finland). Oxygen consumption (VO_2_) and carbon dioxide production (VCO_2_) were measured and REE calculated using the abbreviated Weir formula, REE = [3.9 (VO_2_) + 1.1 (VCO_2_)] 1.44 [2,25]. Measurements were started as soon as infants were not dependent on supplemental oxygen, scheduled twice a week (Tuesdays and Fridays), and performed by the same investigator (SB). Recommended procedures for instrument calibration and measurement technique in small infants were followed [25,32]. Infants were housed either in open cribs or in convective incubators with a servocontrol mechanism regulated by abdominal skin temperature at 36.4 °C to 36.5 °C [4]. The chamber was carefully adapted to prevent air leaks. The activity of the infants was visually assigned every minute during REE measurements using a validated scale [4,33]. In infants receiving intermittent feeding, measurements were undertaken 1-h after bolus meal to avoid the postprandial thermogenesis effect [34]. In infants receiving continuous tube feeding, measurements were performed with no temporal restrictions. Every REE measurement was undertaken during quiet or active sleep, or exceptionally in quiet awake status that has a slightly higher mean REE [4]. Whenever the infant became physically active or agitated, measurements were stopped and repeated later on the same day under resting or sleeping conditions [35]. Measurements were postponed to a subsequent day if axillary temperature was equal to or greater than 37 °C [4]. Each measurement lasted about 40-min, including 10-min for adaptation of the infant to the environment and face chamber [33]. For each 30-min assessment, the continuous period of 20-min of highest quality, without interference of infant movements, was recorded for subsequent analysis.

Measurements were not performed or were temporarily interrupted whenever the infant was unstable on manipulation, in acute phases of sepsis, or if any acute pathological event occurred that might have had a negative impact on the infant’s metabolic condition. Infants were removed from follow-up if they needed further major surgery, transferred to another hospital, or discharged before two-week calorimetry measurements were completed.

The difference between the estimated total energy intake on the day before the indirect calorimetry measurements and the measured REE was considered the energy balance.

### 2.5. Body Composition Assessment

Body composition was assessed by air displacement plethysmography using the Pea Pod equipment (Cosmed, Concord, CA, USA), which measures body mass, and estimates FM and FFM with precision of 0.1 g. Before each body composition assessment, crown-heel length was accurately measured using a standard procedure [36]. The FMI, expressed as FM (kg)/length (m^2^) [37], was used as an indicator of adiposity. For safety reasons, weekly body composition assessments were scheduled as soon as infants were not dependent on supplemental oxygen and only after removal of central venous lines.

### 2.6. Statistical Analysis

An exploratory data analysis was carried out for all variables. Continuous variables not changing over time were described with mean and standard deviation (SD) or median and inter-quantile ranges (IQR) or total ranges (minimum–maximum). Additionally, LOWESS (Locally Weighted Scatterplot Smoothers) plots were used to describe the association between each continuous variable (total energy intake, protein intake, carbohydrate intake, fat intake, and body weight) and postmenstrual age (PMA). For the chronologic record of repeated measurements and calculations, the age after birth (in days), the individual PMA (in complete weeks), and time after surgery (in days) were considered.

Considering that energy and macronutrient needs differ according to the degree of an infant’s maturity [38,39,40,41], and that nutrient support influences REE and RQ [3,7], profiles of total energy intake, macronutrient intakes, REE, RQ, and body weight gain were analyzed separately in infants born preterm (gestational age at birth lower than 37 weeks) and at term (gestational age at birth greater than or equal to 37 weeks). Cross-sectional data for each week PMA on energy and macronutrient intake, REE, energy balance, and RQ were considered only for those PMA weeks that include at least two infants.

To study the association between these variables and the three outcomes of REE, RQ and FMI, univariable and multivariable additive mixed effects regression models were used to consider the correlation structure between measures in time and to allow the modelling of non-linear associations between the outcomes and the independent variables by using spline smoothers. For statistical analysis, weekly total energy and macronutrient intakes on the day before the indirect calorimetry measurements were considered. Crude and adjusted regression coefficients with corresponding 95% confidence intervals were estimated. The level of significance was α = 0.05.

Data analysis was performed using the software Stata (StataCorp. 2013. Stata Statistical Software: Release 13. College Station, TX: StataCorp LP.) and R (R: A Language and Environment for Statistical Computing, R Core Team, R Foundation for Statistical Computing, Vienna, Austria, year = 2019, http://www.R-project.org).

## 3. Results

Thirty-one neonates were initially recruited but two were excluded due to associated congenital heart anomalies complicated with heart failure. Thus, 29 neonates were included for analysis. This sample included 15 infants born preterm (7 females), with a median (minimum–maximum) gestational age of 35.3 (31.4–36.7) weeks and a median birth weight of 2304 (1470–2882) g, and 14 infants born at term (4 females), with a median gestational age of 38.1 (37.1–40.6) weeks and a median birth weight of 2935 (2542–3485) g. All neonates were appropriate for gestational age. The main diagnoses were esophageal atresia (n = 13), gastroschisis (n = 7), duodenal atresia (n = 5), and annular pancreas (n = 4).

No death occurred during or close to the study period and no infant received caffeine or metabolism stimulating drugs during the period of indirect calorimetry assessment.

The individual PMA at which body weight and energy and macronutrient intakes were recorded ranged between 31 weeks gestational age at birth and 47 weeks PMA at discharge.

Indirect calorimetry (REE and RQ) was measured between 34 and 47 weeks PMA, starting at a median (IQR) of 11.8 (7–15) days after surgery. A total of 317 longitudinal indirect calorimetry measurements were performed in the entire sample, 293 (92.4%) during sleep (mostly in deep sleep) and 24 (7.6%) while quietly awake. Forty-five (14.2%) measurements were performed in infants on exclusive parenteral nutrition, 192 (60.6%) 1-h after bolus meal, and 80 (25.2%) in infants on continuous enteral feeding.

The FMI was assessed in a subset of 15 infants, who underwent a total of 22 measurements between 37 and 45 weeks PMA. Calculated FMI is presented for all infants born preterm, but at term PMA, and at term.

Variations of weekly measured total energy intake, macronutrient intake, REE, energy balance, RQ, and body weight gain are presented separately for the cohorts of infants born preterm (n = 15), from 34 to 47 weeks PMA, and those born at term (n = 14), from 38 to 47 weeks PMA.

Data on weekly energy and macronutrient intake, REE, energy balance, and RQ, obtained at 34 weeks and after 42 weeks PMA in infants born preterm, and after 44 weeks PMA in those born at term, included at most two infants and may not be representative of their cohort. Therefore, only values recorded in postmenstrual weeks including more than two infants were considered in the descriptive analysis below.

The median daily total energy intake varied between 80.7 and 109.4 Kcal/kg in infants born preterm, and between 74.0 and 105.2 Kcal/kg in infants born at term. No significant differences were found in energy intake between infants born preterm and at term. Of note, in infants born preterm, during preterm postmenstrual weeks, the median daily total energy intake had been below 91.7 Kcal/kg (Table 1, Figure S1A).

The median daily protein intake varied between 3.0 and 4.0 g/kg in infants born preterm, and between 1.6 to 3.2 g/kg in infants born at term. Infants born preterm had received significantly higher protein intake than those born at term (*p* = 0.049) (Table 2, Figure S1B).

The median daily carbohydrate intake varied between 12.9 and 16.1 g/kg in infants born preterm, and between 10.1 and 15.4 g/kg in infants born at term. No significant differences were found in carbohydrate intake between infants born preterm and at term (Table 3, Figure S1C).

The median daily fat intake varied between 0.8 and 5.2 g/kg in infants born preterm and between 1.1 and 3.8 g/kg in infants born at term. No significant differences were found in fat intake between infants born preterm and at term (Table 4, Figure S1D).

**Figure 1:**
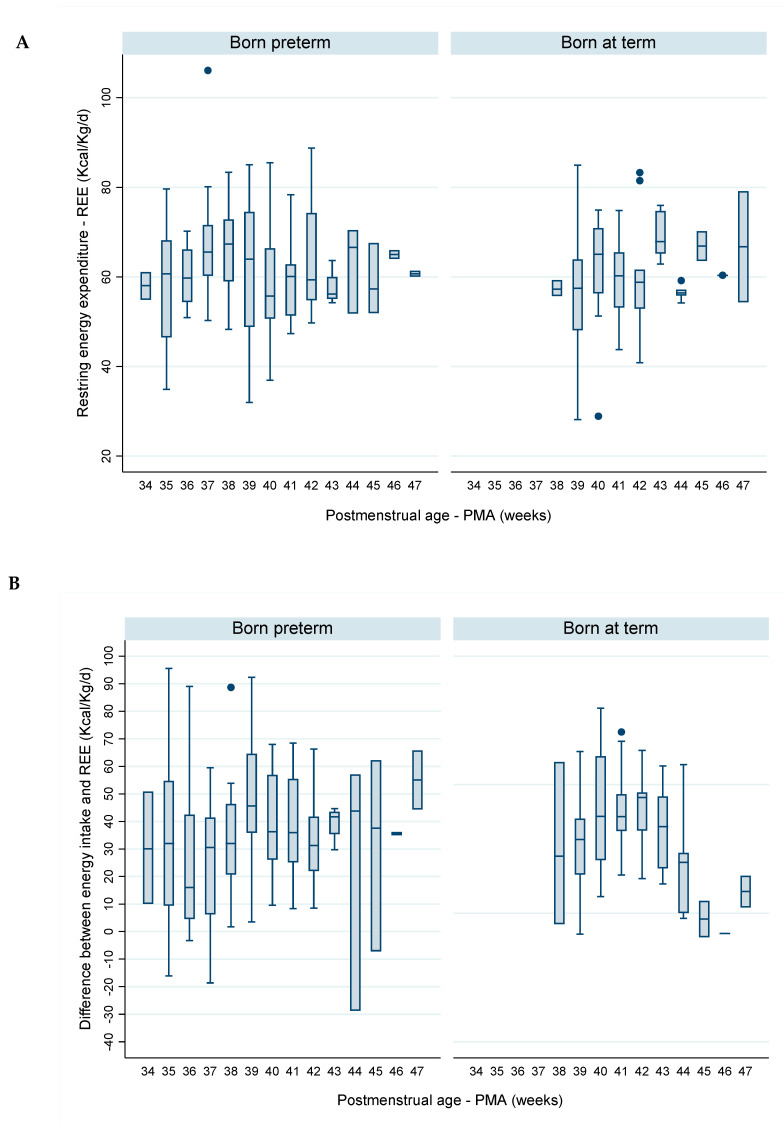

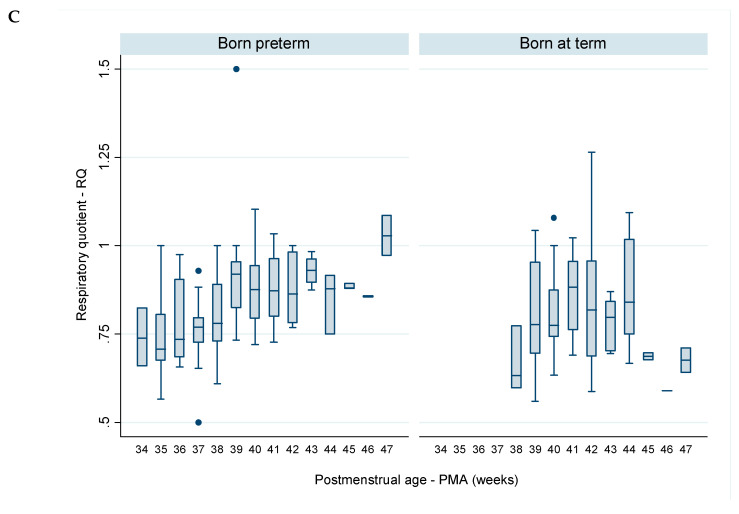
Evolution, per complete week of postmenstrual age (PMA), of (**A**) resting energy expenditure (REE) Kcal/kg/d, (**B**) difference between daily total energy intake and resting energy expenditure (REE), and (**C**) respiratory quotient (RQ), in infants born preterm (n = 15) and at term (n = 14).

The median REE varied between 55.7 and 67.4 Kcal/kg/d in infants born preterm, and between 57.3 and 67.9 Kcal/kg/d in infants born at term (Figure 1A and Table 5). No significant differences were found in REE either between sexes or between preterm and term infants.

The median energy balance was usually positive and varied between 16.1 and 45.6 Kcal/kg/d in infants born preterm and between 19.8 and 45.0 Kcal/kg/d in infants born at term (Figure 1B and Table 6). No significant differences were found in energy balance either between sexes or between infants born preterm and at term.

The median RQ had been usually below 1. The RQ increased from 0.70 at 35 weeks PMA to 0.86–0.92 between 39 and 42 weeks PMA infants born preterm, and from 0.63 at 38 weeks PMA to 0.84–0.88 between 41 and 44 weeks PMA in infants born at term (Figure 1C and Table 7). No significant differences were found either between sexes or between infants born preterm and at term.

Based on daily body weight measurements, the median (IQR) weight gain velocities from birth to discharge was 13.8 (10.7–34.3) g/kg/d in infants born preterm and of 6.0 (2.7–12.9) g/kg/d in infants born at term, being significantly faster in infants born preterm (*p* = 0.037).

**Figure 2:**
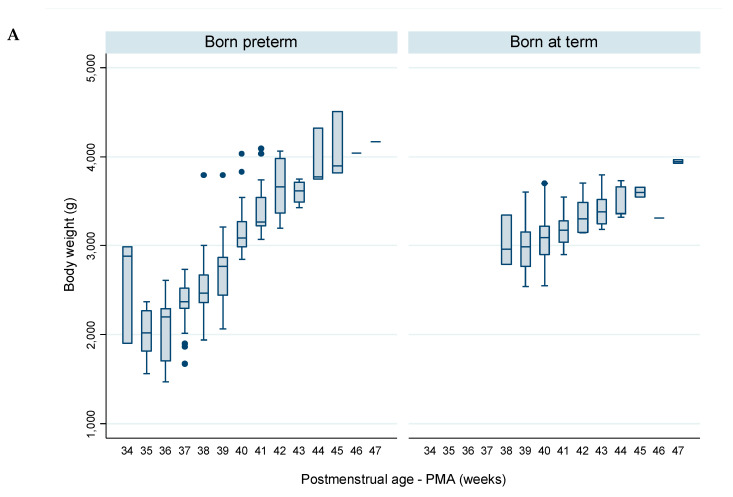

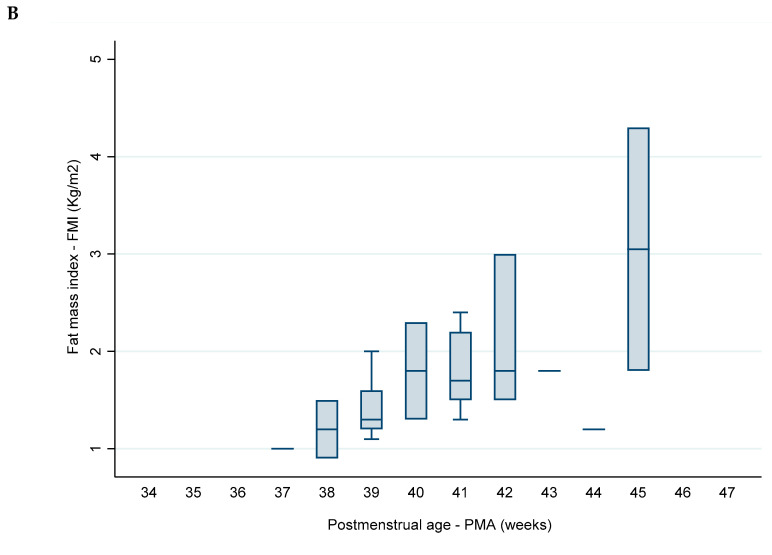
Evolution, per complete week of postmenstrual age, of (**A**) body weight, in infants born preterm (n = 15) and at term (n = 14), and (**B**) fat mass index (FMI), gathering infants born preterm and at term (n = 15).

The variation of weekly body weight, measured on the same days of indirect calorimetry measurements (Figure 2A and Table 8), revealed that:

- In infants born preterm, the median body weight decreased from 2882 g at 34 weeks PMA to 2017 g at 35 weeks PMA, and subsequently a daily increase was observed, reaching 4170 g at 47 weeks PMA.
- In infants born at term, the median body weight increased a daily increase was observed from 2960 g at 38–39 weeks PMA, reaching 3943 g at 47 weeks PMA.

The FMI was assessed in a subset of 15 infants—8 were born preterm and 7 born at term. Following the safety criteria adopted, all infants were measured at or after term PMA. Infants born preterm and at term were analyzed together due to the homogeneity in PMA and the small dimension of the sample. A single measurement was undertaken in twelve infants and two measurements with one-week interval were undertaken in three infants (Figure 2B and Table 9). The median FMI increased from 1.0 kg/m^2^ at 37 weeks PMA to 3.1 kg/m^2^ at 45 weeks PMA.

### Determinants of the Evolution of REE, RQ and Adiposity

Among all infants, preterm and term, no significant univariate associations were found between REE and sex, binary gestational age at birth (born preterm vs. born at term), PMA, weekly measured body weight, or daily total energy intake (Table S1); therefore, no multivariate model was explored for REE as a dependent variable.

Models for determinants of RQ were explored. From univariate analysis (Table S2), PMA, body weight, daily carbohydrate intake, and REE were selected for multivariate analysis, but only PMA, daily carbohydrate intake and REE remained significant in the final model. Accordingly, for each week increase in PMA, there was a mean increase of 0.013 (95% CI: 0.007 to 0.019) in RQ (*p* < 0.001) for infants with the same levels of carbohydrate intake and REE. For each 1 g/kg increase in carbohydrate intake, there was a mean increase of 0.009 (95% CI: 0.004 to 0.014) in RQ (*p* < 0.001) for infants with the same PMA and REE. For each Kcal/kg increase in REE there was a mean decrease of 0.006 (95% CI: −0.007 to −0.005) in RQ (*p* < 0.001) for infants with the same PMA and carbohydrate intake level.

Univariate associations with FMI variation were explored (Table S3). Postmenstrual age, weekly body weight, daily total energy intake, and carbohydrate and fat intakes were identified as candidates for multivariable analysis. Only PMA and daily carbohydrate and fat intakes remained significant in the final multivariate model. Accordingly, a mean increase in FMI of 0.32 kg/m^2^ (95% CI: 0.23 to 0.41; *p* < 0.001) for each additional week of PMA was estimated. A mean decrease in FMI of 0.21 g/m^2^ (95% CI: −3.74 to −0.52; *p* = 0.009) was observed for each additional g/kg in daily carbohydrate intake. A mean increase in FMI of 0.40 g/m^2^ (95% CI: 0.06 to 0.74; *p* = 0.022) was observed for each additional g/kg in daily fat intake.

## 4. Discussion

This cohort study describes the evolution of REE and RQ in infants growing in the late, anabolic phase, after corrective surgery of major congenital gastrointestinal tract anomalies. Factors associated with REE and RQ were analyzed in the entire sample and FMI was analyzed in a subset of preterm and term infants combined, in whom measurements were undertaken at term PMAs.

### 4.1. Body Composition as Major Determinant of Resting Energy Expenditure

Body mass in humans is strongly associated with the rate of heat production, as defined by REE [42]. In healthy adults, the major components of body mass differ in their metabolic rates, estimated as approximately 20 Kcal/kg for FFM and 5 Kcal/kg for FM [43]. The FFM, used as a measure of metabolically active tissue, is quite heterogeneous, comprised of organs and tissues with different cellular components [42,43]. The total body REE is the sum of the individual organ and tissue REEs, and each organ and tissue has unique mass-specific metabolic rate [42]. The contribution of individual organs and tissues to REE vary among age groups. In growing prepubertal children and early adolescents, brain and liver mass have a greater contribution to total body REE than in adults, in whom skeletal muscle mass REE has greater contribution [1,43].

A systematic review had concluded that preterm infants at term equivalent age have greater adiposity and less FFM than those born full-term [44]. As body composition is a major determinant of REE [1,43], it is not surprising that REE of infants born preterm was reported to differ from those born at term, even after term equivalent age [3]. Accordingly, in this study the cohorts of infants born preterm and at term were analyzed separately and compared.

### 4.2. Resting Energy Expenditure, Energy Intake, and Energy Balance

The median REE in infants born preterm varied between 55.7 and 67.4 Kcal/kg/d, from 34 to 42 weeks PMA. In infants born at term, it varied between 57.3 and 67.9 Kcal/kg/d from 38 to 44 weeks PMA, not differing significantly from those born preterm. Available REE reference values described for healthy infants showed higher mean values in preterm infants (29–32 weeks of gestation), increasing from 46 to 81 Kcal/kg/d during the first six postnatal weeks, compared to the increase from 42 to 62 Kcal/kg/d in term infants [3]. Data on REE in neonates undergoing major surgery are scarce, and probably specific for the type of surgical condition [14,15,17] and for the postsurgical period (early or late) [6,9,13]. In our study, no significant differences in REE were found between preterm and term infants, probably in part because these preterm infants were predominantly late preterm. No multivariate regression analysis model was found for REE and the small sample size in our study may be contributory to this lack of difference.

Suboptimal nutritional support is usual following major surgery of gastrointestinal tract, due to non-physiological feeding (commonly only intravenous and then only slow advancement of often dilute enteral feeding), as well as and increased protein synthesis for tissue repair, which is a high energy-requiring process [21]. In our study, we found that the median energy intake was frequently below or close to the minimum 90 Kcal/kg/d recommended for parenteral and enteral nutrition [38,39,45,46]; median energy intakes in infants born preterm varied between 80.7 and 115.8 Kcal/kg/d, and in infants born at term between 64.6 and 105.2 Kcal/kg/d. This resulted in periods of low energy balance, which varied between a median of 16.1 and 41.6 Kcal/kg/d in infants born preterm and 19.8 and 45.0 Kcal/kg/d in those born at term. These values are insufficient to cover energy cost of growth (tissue synthesis and energy stores), which is estimated to be 50–70 Kcal/kg/d in a typical term surgical neonate [9,47].

### 4.3. Respiratory Quotient and Macronutrients Intake

In infants born preterm, the median RQ increased from 0.70–0.74 at 35 weeks PMA to 0.86–0.92 between 39 and 42 weeks PMA, similar to infants born at term, in which it increased from 0.63 at 38 weeks PMA to 0.80–0.88 between 41 and 44 weeks PMA.

Physiological human RQ values are within a range of 0.67–1.2, depending on the balance of different substrates with different RQ values [7]: values close to 0.67–0.69 indicate underfeeding and fat oxidation, those close to 0.81–0.82 indicate protein oxidation, those at ~0.85 indicate mixed substrate oxidation, that at 1.0 indicate glucose oxidation, and those between 1.0 and 1.2 indicate overfeeding and lipogenesis [7,25].

Overall, the median protein and carbohydrate intakes in the entire sample reached the minimum recommended [38,40,45]. Infants born preterm received median fat intake between 0.8 and 5.2 g/kg/d; in some postmenstrual weeks it was much lower than the minimum recommended of 2.5 g/kg/d by parenteral nutrition and of 4.8 g/kg/d by enteral nutrition [45]. The median fat intake in infants born at term was as low as 1.1 to 1.3 g/kg/d at 38–39 weeks PMA, although most received sufficient fat intake [38].

According to multivariate regression analysis, increase in RQ was associated with an increase in PMA for infants with the same levels of carbohydrate intake and REE, and was associated with an increase in carbohydrates intake for infants with the same PMA and levels of REE. Decrease in RQ was associated with increase in REE for infants with the same PMA and carbohydrate intake. Interpreting these associations, carbohydrate intake (promoting glucose oxidation) is well-known to correlate positively with the RQ, including in preterm infants [11]. Contrary to the increase in RQ with postnatal age that we found in our study, no significant RQ changes were reported in non-surgical preterm neonates for three weeks after achieving full enteral feedings [48]. In our infants, the increase in RQ with postnatal age may be related with body weight gain occurring in the presence of sufficient carbohydrate intake, even if associated with insufficient fat intake. With advancement in postnatal age, energy cost of growth to support body mass gain may have been derived from carbohydrates, reflected by an increase in RQ due to glucose oxidation. We speculate that the association between the RQ decrease and REE increase may be explained by some degree of lipolysis suggested by frequent RQ values around or below 0.8. In an animal model, it was found that increasing lipolysis in adipose tissue caused a shift within adipocytes towards increased fatty acid utilization accompanied by increase in energy expenditure that limited the release of fatty acids into the circulation for oxidation elsewhere in the body but protected against adiposity [49].

### 4.4. Body Weight Gain and Adiposity Catch-Up

In spite of some deficit in energy, after an initial body weight decrease, a continuous increase was recorded from birth to discharge, with satisfactory median weight gain velocity in infants born preterm. The median weight gain velocity in infants born at term was significantly slower than in those born preterm and slower than reported for healthy term infants [50].

To confirm the previously reported low adiposity in spite of satisfactory body weight gain described in infants with similar surgical conditions [16], the present study was focused on adiposity rather than other body composition measurements. As an indicator of adiposity, FMI was preferred in relation to FM percentage (%FM), because %FM is a ratio with FM included both in the numerator and denominator (as a component of body mass) [51]. Adjusting FM to a measure unrelated to body fat, such as body length, defines excess adiposity better than FM% [51]. This has been shown in preterm infants [37]. Adiposity (FMI) was analyzed in a combined group of 15 preterm and term infants at term PMA, due to our small sample size, despite prior evidence that adiposity may differ between preterm infants at term equivalent age and infants born full-term [44]. The FMI increased from a median of 1.0 kg/m^2^ at 37 weeks PMA to a median of 1.7 to 1.8 Kg/m^2^ at 40 to 43 weeks PMA. These values are lower than the reference median value of 2.5 Kg/m^2^ at 40 weeks PMA reported in non-surgical infants that were assessed using dual-energy X-ray absorptiometry [37]. The low adiposity we found is consistent with poor energy balance, low fat intake, and RQ frequently around 0.8 or lower, indicating increased fat oxidation.

If body composition had been measured concurrently with indirect calorimetry during the entire study period, it would have provided a better interpretation of changes in REE and RQ, since FFM is a surrogate of metabolically active tissue and a major determinant of REE [42,43]. For safety reasons, body composition was assessed only after the removal of central catheters and oxygen use. Consequently, measurements were undertaken late and close to discharge only in approximately half of the infants, with most of the infants having only a single body composition measurement. For this reason, it was not possible to construct multivariate regression models to explain REE, with FFM, FM, and FMI as independent variables. Instead, a multivariate regression model was constructed to explain adiposity, with REE as an independent variable. Accordingly, an increase in FMI was associated with both greater PMA and fat intake, and a decrease in FMI was associated with greater carbohydrate intake. The first associations were expected, since the FMI catch-up occurred with increase in postnatal age and corresponds to lipogenesis, for which fat intake is fundamental. We speculate that the FMI decrease is associated with greater carbohydrates intake, especially in infants born at term, may be a consequence of periods of low lipogenesis or even lipolysis due to preferential fat oxidation (indicated by low RQ). This may have resulted from low overall energy intake and poor energy balance along with the low fat intake [38], despite fact that the protein and carbohydrate intakes were in the range considered to be sufficient [38,40,45].

To the best of our knowledge, this is the first cohort study describing longitudinal variations in REE and RQ after corrective surgery of major congenital gastrointestinal tract anomalies. Combining indirect calorimetry with body composition assessment is a strength of the study, because it provided more accurate information of effects of energy and macronutrients intake on the nutritional status of the infants studied [28]. Previously, both approaches have been used in studies only by Pereira-da-Silva et al. (2015) [16] in infants with similar surgical conditions to those herein studied and by Irving et al. (2013) [15] who studied infants subjected to surgery for congenital heart disease.

Some limitations of this study should be acknowledged. First, the sample size may be too small to allow enough statistical power for some of the associations tested. Second, in approximately a quarter of the infants, the REE was measured while infants were receiving continuous enteral feeding. Although this produces some thermogenesis effect, in small infants this effect has been reported to be much lower than after a bolus feeding [52]. Third, body composition was measured in only approximately half of the sample and at advanced PMAs; therefore, it may not represent the entire sample.

## 5. Conclusions

This cohort study describes the evolution of REE and RQ in infants growing during the late anabolic phase, after corrective surgery of major congenital gastrointestinal tract anomalies. In infants born preterm, the median REE varied between 55.7 and 67.4 Kcal/kg/d and the median RQ increased from 0.70 to 0.86–0.92, from 34 to 42 weeks PMA. In those born at term, the median REE varied between 57.3 and 67.9 Kcal/kg/d and the median RQ increased from 0.63 to 0.84–0.88, from 38 to 44 weeks PMA. Despite some deficit in energy, continuous body weight gain was recorded, with satisfactory weight gain velocity in infants born preterm, but slower than reported for healthy infants in those born at term. Adiposity was measured at late PMA in approximately in half of the infants studied. At term PMA, the median FMI was between 1.7 and 1.8 kg/m^2^, lower than reported in non-surgical infants. This low adiposity is consistent with several study periods in which low intake of energy was observed, during which there was increased fat oxidation as indicated by the low RQ.

Data from this study indicate that after the early phase of corrective surgery of major congenital gastrointestinal tract anomalies, higher energy and fat intakes are needed to cover energy cost of growth and reduce fat oxidation, thus preventing lipolysis and promoting lipogenesis, particularly in term infants. Further studies are needed to confirm these results and improve the nutritional approach in this population.

## Figures and Tables

**Table 1:** Daily energy intake in infants born preterm and at term.

Born Preterm (n = 15)														
Postmenstrual age (weeks)	34	35	36	37	38	39	40	41	42	43	44	45	46	47
Individuals (n)	2	4	7	11	12	13	8	8	6	2	2	2	1	1
Measurements (n)	3	8	11	21	24	19	16	14	11	4	3	3	2	2
Median (Kcal/kg)	88.1	90.4	80.7	91.7	98.8	109.4	96.7	93.2	93.2	97.8	108.9	89.5	100.6	115.8
Percentile 25 (Kcal/kg)	71.1	79.2	65.9	71.8	83.8	91.7	83.8	90.1	87.5	95.4	41.8	60.4	99.0	104.3
Percentile 75 (Kcal/kg)	105.8	102.8	98.1	107.7	107.7	119.7	112.5	106.8	106.7	98.6	110.4	119.5	102.2	127.2
Born at Term (n = 14)														
Postmenstrual age (weeks)	38	39	40	41	42	43	44	45	46	47				
Individuals (n)	3	9	11	9	7	5	4	2	1	1				
Measurements (n)	3	12	19	14	11	7	5	2	1	2				
Median (Kcal/kg)	79.5	82.2	96.4	99.4	105.2	99.5	74.0	64.6	52.5	75.2				
Percentile 25 (Kcal/kg)	51.4	78.5	90.7	90.8	93.5	81.6	55.9	54.2	52.5	68.9				
Percentile 75 (Kcal/kg)	118.1	90.9	113.5	108.6	108.8	121.4	79.8	75.0	52.5	81.4				

**Table 2:** Daily protein intake in infants born preterm and at term.

Born Preterm (n = 15)														
Postmenstrual age (weeks)	34	35	36	37	38	39	40	41	42	43	44	45	46	47
Individuals (n)	2	4	7	11	12	13	8	8	6	2	2	2	1	1
Measurements (n)	3	8	11	21	24	19	16	14	11	4	3	3	2	2
Median (g/kg)	3.4	4.0	3.0	3.3	3.1	3.4	3.6	3.6	3.6	3.6	3.4	3.1	3.6	4.5
Percentile 25 (g/kg)	3.4	3.4	2.8	3.0	2.4	2.7	3.1	3.1	3.3	3.3	1.3	1.1	3.6	3.9
Percentile 75 (g/kg)	4.0	4.3	3.8	3.6	3.6	3.9	3.7	3.8	3.7	3.8	3.9	4.0	3.7	5.0
Born at term (n = 14)														
Postmenstrual age (weeks)	38	39	40	41	42	43	44	45	46	47				
Individuals (n)	3	9	11	9	7	5	4	2	1	1				
Measurements (n)	3	12	19	14	11	7	5	2	1	2				
Median (g/kg)	3.2	3.0	3.0	3.2	3.0	2.5	1.6	1.4	1.1	3.8				
Percentile 25 (g/kg)	2.6	2.8	2.0	2.3	2.5	2.2	1.3	1.1	1.1	3.6				
Percentile 75 (g/kg)	5.3	3.4	3.5	3.7	3.4	3.3	2.1	1.7	1.1	4.0				

**Table 3:** Daily carbohydrate intake in infants born preterm and at term.

Born Preterm (n = 15)														
Postmenstrual age (weeks)	34	35	36	37	38	39	40	41	42	43	44	45	46	47
Individuals (n)	2	4	7	11	12	13	8	8	6	2	2	2	1	1
Measurements (n)	3	8	11	21	24	19	16	14	11	4	3	3	2	2
Median (g/kg)	13.2	15.3	14.0	12.9	13.6	14.8	14.5	16.0	16.1	16.1	14.5	13.0	15.3	20.6
Percentile 25 (g/kg)	12.6	15.0	10.0	11.9	11.7	12.1	13.1	13.0	14.8	15.7	3.0	6.9	15.2	18.4
Percentile 75 (g/kg)	13.3	16.5	14.7	15.1	16.2	16.6	16.2	16.9	17.3	17.1	16.4	16.8	15.5	22.7
Born at term (n = 14)														
Postmenstrual age (weeks)	38	39	40	41	42	43	44	45	46	47				
Individuals (n)	3	9	11	9	7	5	4	2	1	1				
Measurements (n)	3	12	19	14	11	7	5	2	1	2				
Median (g/kg)	13.3	14.4	13.4	15.4	13.8	13.8	10.1	8.6	6.7	13.6				
Percentile 25 (g/kg)	9.0	12.8	10.6	11.8	12.8	13.2	7.1	6.9	6.7	12.5				
Percentile 75 (g/kg)	21.7	15.3	17.4	16.9	14.8	15.4	13.2	10.3	6.7	14.7				

**Table 4:** Daily fat intake in infants born preterm and at term.

Born Preterm (n = 15)														
Postmenstrual age (weeks)	34	35	36	37	38	39	40	41	42	43	44	45	46	47
Individuals (n)	2	4	7	11	12	13	8	8	6	2	2	2	1	1
Measurements (n)	3	8	11	21	24	19	16	14	11	4	3	3	2	2
Median (g/kg)	2.1	1.3	0.8	2.6	3.6	5.2	1.5	1.3	1.3	1.9	3.1	3.1	2.7	1.7
Percentile 25 (g/kg)	0.8	0.8	0.8	0.8	1.1	1.8	0.9	1.0	0.9	1.6	2.8	2.7	2.6	1.7
Percentile 75 (g/kg)	4.3	2.4	6.1	4.8	5.6	7.9	5.5	5.5	3.2	2.2	4.3	4.0	2.9	1.8
Born at term (n = 14)														
Postmenstrual age (weeks)	38	39	40	41	42	43	44	45	46	47				
Individuals (n)	3	9	11	9	7	5	4	2	1	1				
Measurements (n)	3	12	19	14	11	7	5	2	1	2				
Median (g/kg)	1.1	1.3	3.8	3.1	3.6	5.2	2.9	2.8	2.4	0.6				
Percentile 25 (g/kg)	0.6	0.8	2.6	1.3	1.5	0.7	2.5	2.5	2.4	0.5				
Percentile 75 (g/kg)	1.5	2.9	4.8	5.1	5.8	6.4	3.0	3.0	2.4	0.7				

**Table 5:** Evolution of resting energy expenditure in infants born preterm and at term.

Born Preterm (n = 15)														
Postmenstrual age (weeks)	34	35	36	37	38	39	40	41	42	43	44	45	46	47
Individuals (n)	2	4	7	11	12	13	8	8	6	2	2	2	1	1
Measurements (n)	3	8	11	21	24	19	16	14	11	4	3	3	2	2
Median (Kcal/kg/d)	58.1	60.7	59.8	65.6	67.4	64.0	55.7	60.1	59.4	56.2	66.6	57.3	65.0	60.7
25th Percentile (Kcal/kg/d)	54.9	46.5	54.4	60.2	59.0	48.9	50.7	51.3	54.8	55.1	51.8	51.9	64.0	60.1
75th Percentile (Kcal/kg/d)	61.1	68.2	66.2	71.6	72.8	74.5	66.4	62.8	74.3	60.0	70.5	67.6	66.0	61.4
Born at term (n = 14)														
Postmenstrual age (weeks)	38	39	40	41	42	43	44	45	46	47				
Individuals (n)	3	9	11	9	7	5	4	2	1	1				
Measurements (n)	3	12	19	14	11	7	5	2	1	2				
Median (Kcal/kg/d)	57.3	57.5	65.1	60.2	58.8	67.9	56.4	66.9	60.4	66.7				
25th Percentile (Kcal/kg/d)	55.7	48.1	56.3	53.2	52.9	65.2	55.8	63.6	60.4	54.3				
75th Percentile (Kcal kg/d)	59.3	63.9	70.9	65.5	61.6	74.7	57.2	70.2	60.4	79.2				

**Table 6:** Evolution of energy balance (total energy intake minus REE) in infants born preterm and at term REE, resting energy expenditure.

Born Preterm (n = 15)														
Postmenstrual age (weeks)	34	35	36	37	38	39	40	41	42	43	44	45	46	47
Individuals (n)	2	4	7	11	12	13	8	8	6	2	2	2	1	1
Measurements (n)	3	8	11	21	24	19	16	14	11	4	3	3	2	2
Median (Kcal/kg/d)	30.1	32.0	16.1	30.6	32.0	45.6	36.2	35.9	31.3	41.6	43.8	37.5	35.6	55.0
25th Percentile (Kcal/kg/d)	10.0	9.4	4.6	6.2	20.7	35.8	26.1	25.1	22.0	35.3	−28.8	−7.2	35.0	44.3
75th Percentile (Kcal kg/d)	50.9	54.7	42.4	41.4	46.4	64.5	56.9	55.5	41.8	43.5	57.0	62.2	36.2	65.8
Born at term (n = 14)														
Postmenstrual age (weeks)	38	39	40	41	42	43	44	45	46	47				
Individuals (n)	3	9	11	9	7	5	4	2	1	1				
Measurements (n)	3	12	19	14	11	7	5	2	1	2				
Median (Kcal/kg/d)	22.2	28.7	37.6	37.5	45.0	33.7	19.8	−2.3	−7.8	8.4				
25th Percentile (Kcal kg/d)	−4.3	15.1	20.6	31.9	32.2	17.5	0.0	−9.4	−7.8	2.3				
75th Percentile (Kcal kg/d)	58.8	36.8	61.1	46.3	47.0	45.5	23.5	4.8	−7.8	14.6				

**Table 7:** Evolution of respiratory quotient in infants born preterm and at term.

Born Preterm (n = 15)														
Postmenstrual age (weeks)	34	35	36	37	38	39	40	41	42	43	44	45	46	47
Individuals (n)	2	4	7	11	12	13	8	8	6	2	2	2	1	1
Measurements (n)	3	8	11	21	24	19	16	14	11	4	3	3	2	2
Median (g/kg/d)	0.74	0.70	0.74	0.77	0.78	0.92	0.88	0.87	0.86	0.93	0.88	0.88	0.86	1.03
25th Percentile (g/kg/d)	0.66	0.67	0.68	0.73	0.73	0.82	0.79	0.80	0.78	0.90	0.75	0.88	0.85	0.97
75th Percentile (Kcal/kg/d)	0.82	0.81	0.90	0.80	0.89	0.95	0.94	0.96	0.98	0.96	0.92	0.90	0.86	1.09
Born at term (n = 14)														
Postmenstrual age (weeks)	34	35	36	37	38	39	40	41	42	43	44	45	46	47
Individuals (n)	3	9	11	9	7	5	4	2	1	1				
Measurements (n)	3	12	19	14	11	7	5	2	1	2				
Median (g/kg/d)	0.63	0.78	0.77	0.88	0.82	0.80	0.84	0.69	0.59	0.68				
25th Percentile (g/kg/d)	0.60	0.70	0.74	0.76	0.69	0.70	0.75	0.68	0.59	0.64				
75th Percentile (Kcal/kg/d)	0.77	0.95	0.87	0.96	0.96	0.84	1.02	0.70	0.59	0.71				

**Table 8:** Evolution of body weight in infants born preterm and at term.

Born Preterm (n = 15)														
Postmenstrual age (weeks)	34	35	36	37	38	39	40	41	42	43	44	45	46	47
Individuals (n)	2	4	7	11	12	13	8	8	6	2	2	2	1	1
Measurements (n)	3	8	11	21	24	19	16	14	11	4	3	3	2	2
Median (g)	2882	2017	2200	2370	2468	2765	3085	3265	3660	3618	3775	3900	4040	4170
25th Percentile (g)	1905	1811	1700	2290	2360	2445	2985	3219	3365	3490	3750	3820	4040	4170
75th Percentile (g)	2990	2270	2290	2522	2672	2870	3272	3540	3980	3715	4320	4505	4040	4170
Born at term (n = 14)														
Postmenstrual age (weeks)	38	39	40	41	42	43	44	45	46	47				
Individuals (n)	3	9	11	9	7	5	4	2	1	1				
Measurements (n)	3	12	19	14	11	7	5	2	1	2				
Median (g)	2960	2990	3090	3175	3300	3380	3360	3600	3310	3943				
25th Percentile (g)	2790	2762	2900	3040	3150	3245	3355	3540	3310	3920				
75th Percentile (g)	3345	3155	3220	3280	3485	3520	3660	3659	3310	3965				

**Table 9:** Evolution of adiposity indicated by fat mass index (FMI) (kg/m^2^) after term postmenstrual age, in a subset of infants (n = 15) born preterm and at term.

Postmenstrual Age (Weeks)	37	38	39	40	41	42	43	44	45
Measurements (n)	1	2	5	2	5	3	1	1	2
Median (kg/m^2^)	1.0	1.2	1.3	1.8	1.7	1.8	1.8	1.2	3.1
25th Percentile (kg/m^2^)	1.0	0.9	1.2	1.3	1.5	1.5	1.8	1.2	1.8
75th Percentile (kg/m^2^)	1.0	1.5	1.6	2.3	2.2	3	1.8	1.2	4.3

## Supplementary Materials

The following are available online at https://www.mdpi.com/2072-6643/12/10/3093/s1, Table S1. Univariable regression analysis, dependent variable: weekly resting energy expenditure, Table S2. Univariable regression analysis, dependent variable: respiratory quotient, and Table S3. Univariable regression analysis, dependent variable: FMI. Figure S1. Variation, per complete week of postmenstrual age, of daily intakes of (1A) total energy Kcal/kg, (1B) protein g/kg, (1C) carbohydrates g/kg, and (1D) fat g/kg in infants born preterm (n = 15) and at term (n = 14).

## Abbreviations

%FM	fat mass percentage
FFM	fat-free mass
FM	fat mass
PMA	postmenstrual age
REE	resting energy expenditure
RQ	respiratory quotient
