# Supplementary material for: Evolution of Resting Energy Expenditure, Respiratory Quotient, and Adiposity in Infants Recovering from Corrective Surgery of Major Congenital Gastrointestinal Tract Anomalies: A Cohort Study
Source: Nutrients. 2020 Oct 11;12(10):3093. doi: 10.3390/nu12103093 (PMC7599456; doi:10.3390/nu12103093)
Supplement: Supplementary file 1 [file nutrients-12-03093-s001.pdf]

Supplementary material

Table S1. Univariable regression analysis, dependent variable: weekly resting energy expenditure.

Model	Coefficient estimate	95% CI		p-value
Sex†	-1.48	-5.54	2.59	0.476
Gestational age at birth††	-1.73	-5.85	2.39	0.412
Postmenstrual age (weeks)	0.30	-0.32	0.92	0.348
Weekly body weight (Kg)	-1.17	-3.96	1.62	0.411
Daily total energy intake (Kcal/Kg)	0.06	-0.02	0.13	0.141

†Reference category: female; ††Reference category: born preterm; CI: confidence interval; p-values were obtained by generalized linear mixed effects regression models.

Supplementary material

Table S2. Univariable regression analysis, dependent variable: respiratory quotient.

Model	Coefficient estimate	95% CI		p-value
Sex†	0.01	-0.04	0.06	0.787
Gestational age at birth††	-0.01	-0.01	0.04	0.640
Postmenstrual age (weeks)†††	0.06	-0.00	0.11	0.057
Body weight (kg)	0.06	0.02	0.09	<0.001
Daily total energy intake (kcal/kg)	0.00	0.00	0.00	0.053
Daily protein intake (g/kg)	0.01	-0.01	0.03	0.291
Daily carbohydrates intake (g/kg)	0.01	0.00	0.01	0.006
Daily fat intake (g/kg)	0.00	-0.01	0.01	0.784
REE (kcal/kg)	-0.01	-0.01	0.00	<0.001

†Reference category: female; ††Reference category: born preterm; †††Reference category: preterm corrected gestational age; CI: confidence interval; REE: resting energy expenditure; p-values were obtained by generalized linear mixed effects regression models.

Supplementary material

Table S3. Univariable regression analysis, dependent variable: FMI.

Model	Coefficient estimate	95% CI		p-value
Sex†	-0.36	-1.17	0.45	0.380
Gestational age at birth††	-0.18	-0.95	0.59	0.648
Postmenstrual age (weeks)	0.28	0.18	0.37	<0.001
Body weight (Kg)	1.04	0.69	1.41	<0.001
Daily total energy intake (Kcal/Kg)	-0.01	-0.03	0.00	0.126
Daily protein intake (g/Kg)	-0.05	-0.40	0.30	0.777
Daily carbohydrates intake (g/Kg)	-1.27	-2.70	0.15	0.080
Daily fat intake (g/Kg)	-0.20	-0.48	0.08	0.153
Respiratory quotient	0.26	-0.72	1.24	0.606

†Reference category: female; ††Reference category: born preterm; CI: confidence interval; p-values were obtained by generalized linear mixed effects regression models.

Supplementary material

1A

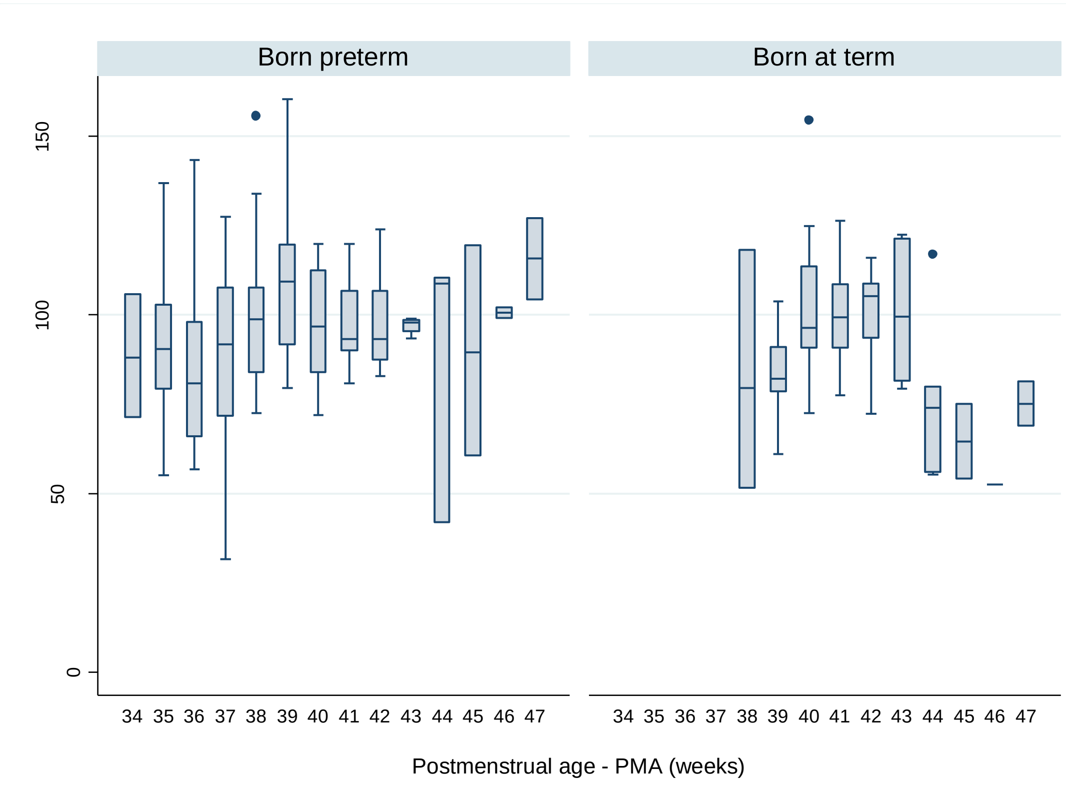

1B

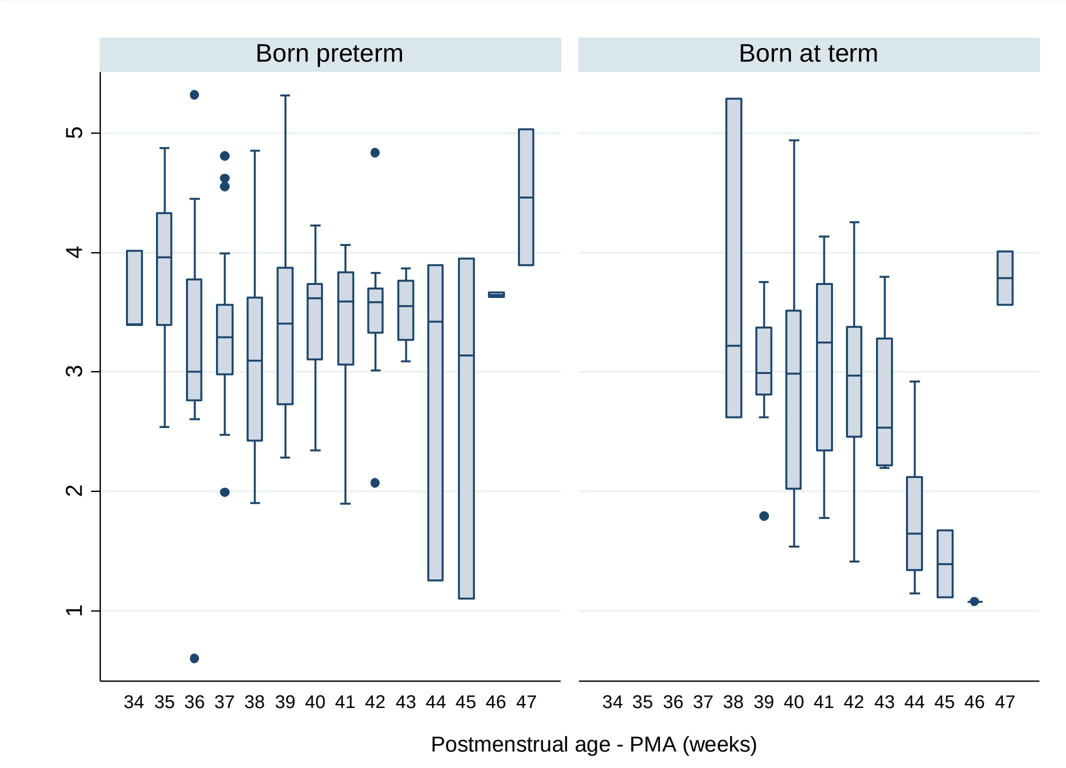

Supplementary material

1C

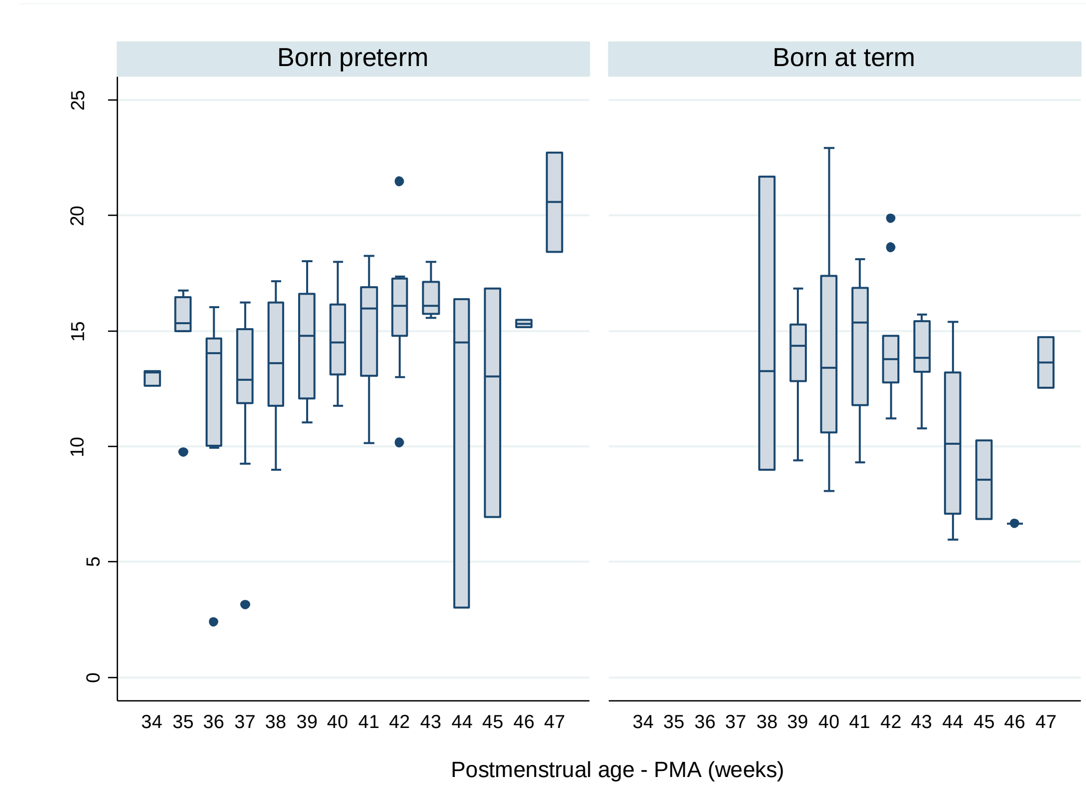

1D

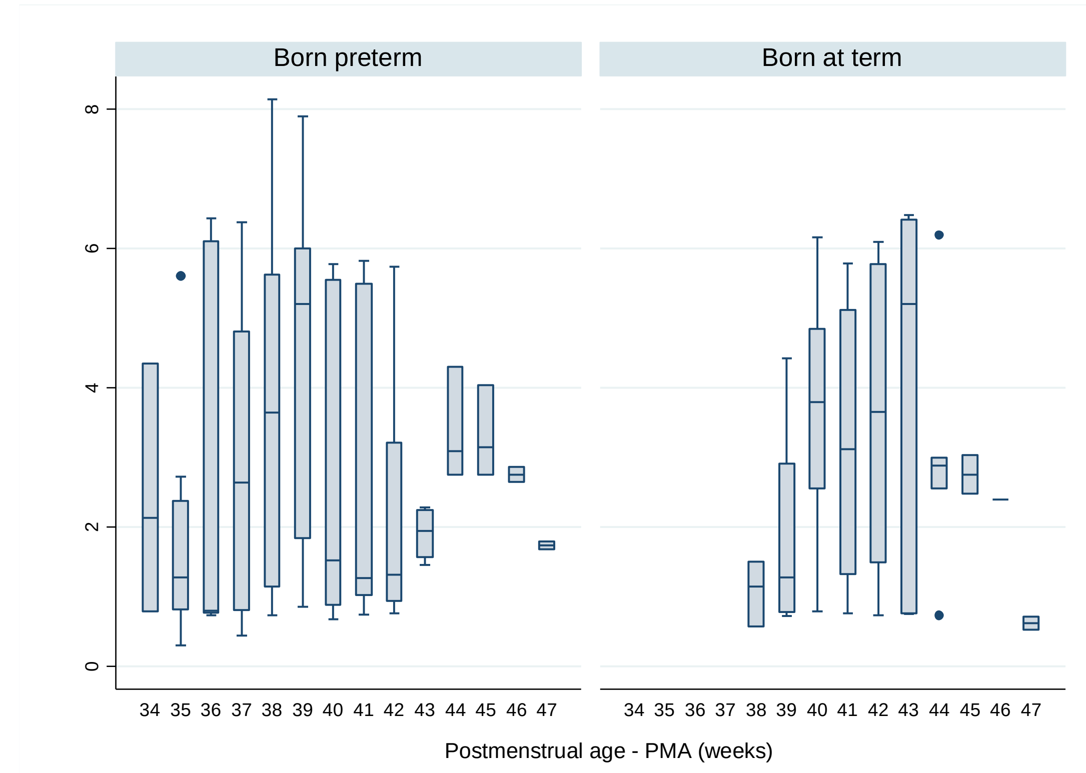

Figure S1. Variation, per complete week of postmenstrual age, of daily intakes of (1A) total energy Kcal/kg, (1B) protein g/kg, (1C) carbohydrates g/kg, and (1D) fat g/kg in infants born preterm (n=15) and at term (n=14).
